# Ontogeny of the Alligator Cartilago Transiliens and Its Significance for Sauropsid Jaw Muscle Evolution

**DOI:** 10.1371/journal.pone.0024935

**Published:** 2011-09-16

**Authors:** Henry P. Tsai, Casey M. Holliday

**Affiliations:** Integrative Anatomy, Department of Pathology and Anatomical Sciences, University of Missouri, Columbia, Missouri, United States of America; University of Maryland, United States of America

## Abstract

The cartilago transiliens is a fibrocartilaginous structure within the jaw muscles of crocodylians. The cartilago transiliens slides between the pterygoid buttress and coronoid region of the lower jaw and connects two muscles historically identified as m. pseudotemporalis superficialis and m. intramandibularis. However, the position of cartilago transiliens, and its anatomical similarities to tendon organs suggest the structure may be a sesamoid linking a single muscle. Incompressible sesamoids often form inside tendons that wrap around bone. However, such structures rarely ossify in reptiles and have thus far received scant attention. We tested the hypothesis that the cartilago transiliens is a sesamoid developed within in one muscle by investigating its structure in an ontogenetic series of *Alligator mississippiensis* using dissection, 3D imaging, and polarizing and standard light microscopy. In all animals studied, the cartilago transiliens receives collagen fibers and tendon insertions from its two main muscular attachments. However, whereas collagen fibers were continuous within the cartilaginous nodule of younger animals, such continuity decreased in older animals, where the fibrocartilaginous core grew to displace the fibrous region. Whereas several neighboring muscles attached to the fibrous capsule in older individuals, only two muscles had significant contributions to the structure in young animals. Our results indicate that the cartilago transiliens is likely a sesamoid formed within a single muscle (i.e., m. pseudotemporalis superficialis) as it wraps around the pterygoid buttress. This tendon organ is ubiquitous among fossil crocodyliforms indicating it is a relatively ancient, conserved structure associated with the development of the large pterygoid flanges in this clade. Finally, these findings indicate that similar tendon organs exist among potentially homologous muscle groups in birds and turtles, thus impacting inferences of jaw muscle homology and evolution in sauropsids in general.

## Introduction

Sesamoids are organized, incompressible structures that often form inside portions of tendons that wrap around bony protuberances. Sesamoids are ubiquitous among vertebrates, are composed of a suite of connective tissues, and vary in their degree of calcification, ranging from osseous (e.g. the mammalian patella) to fibrocartilaginous (e.g. the olecranon sesamoid of lizards, [1]). The incompressible nature of these tissues prevents tendon flattening and increases a muscle's mechanical advantage by lengthening the moment arm of the tendon as it passes around the bone. Sesamoids occur under a wide range of developmental environments. Some sesamoids form as plastic responses to externally applied compression (e.g. the mammalian fabella), whereas others, such as the human patella and retrocalcaneal sesamoid fibrocartilage, form during embryonic development, well before the onset of rigorous, externally applied loads [2], [3]. These studies indicate that formation of sesamoid structures, whether genetic or epigenetic, is intimately associated with mechanical demand for muscle tendons to withstand compression during life.

Fibrocartilaginous sesamoids contain collagen fibers, which resist axial tensional force from the tendon, and a cartilaginous matrix, which resists compressive loads as the tendon presses against bone [4]. However, unlike the parallel arrangement of collagen fibers in tendons, collagen fibers within fibrocartilaginous sesamoids tends to be interwoven within the cartilaginous matrix, an arrangement which has been demonstrated in-vitro to resist compressive loads [5]. In addition, the cartilaginous matrix of are rich in glycosaminoglycan and aggrecan, both which aid in retention of water, thus contributing to the incompressible nature of fibrocartilaginous sesamoids [6]. Whereas sesamoids tend to ossify in limb elements of mammals and birds and calcify in lepidosaurs [7], [8], sesamoids persist as fibrocartilaginous structures in the limb musculature of turtles and crocodylians [9]. However, little attention has been paid to fibrocartilaginous sesamoids in the cranial musculature of vertebrates [9].

The cartilago transiliens (etymology: leaping from one to another) is a cartilaginous junction found in the jaw muscles of crocodylians [10], [11] and turtles [12]. Both cartilaginous structures are similarly named yet occur in markedly different jaw muscles and are clearly non-homologous structures. Partially encased by a fibrous sheath, the crocodylians cartilago transiliens lies between the pterygoid buttress and the mandible, providing significant attachment sites for m. pseudotemporalis superficialis dorsally and m. intramandibularis ventrally, with some contributing attachments from other surrounding muscles. Among sauropsids, m. intramandibularis is present in turtles, crocodylians, and birds [13]. Using *in vivo* electromyography and X-ray imaging of jaw movement during the feeding process of *Caiman crocodilus*, Dullemeijer and Van Drongelen [14] suggested the cartilago transiliens serves as a jaw-locking mechanism. Holliday and Witmer [13] hypothesized that the cartilago transiliens is a sesamoid formed within m. pseudotemporalis superficialis as it wraps around the large, descending pterygoid buttress of crocodylians.

Here we investigate the gross anatomy, microstructure, and ontogeny of the cartilago transiliens in the American alligator (*Alligator mississippiensis*). We tested whether the cartilago transiliens exhibits the characteristics of a sesamoid, and whether its two main muscle attachments, mm. pseudotemporalis superficialis (mPSTs) and intramandibularis (mIRA), are the same muscle. If the cartilago transiliens is a sesamoid, it should exhibit characteristics such as a cartilaginous core with interweaving collagen fibers, articulation with a bony trochlea, and location within a continuous muscle or tendon during development. Furthermore, if mPSTs and mIRA are actually one muscle, fibers from the two muscles should be continuous within the sesamoid's matrix during early ontogeny. Anatomical abbreviations are listed in Table 1.

**Table 1:** Anatomical abbreviations.

**V_3_**	mandibular nerve
**cc**	coronoid fibrocartilage
**ccf**	continuous collagen fibers
**ce**	coronoid eminence
**cr**	cartilaginous (nodular) region
**ct**	cartilago transiliens
**de**	dentary bone
**fs**	fibrous sheath
**icf**	internal collagen fibers
**ju**	jugal bone
**mAMEM**	m. adductor mandibulae externus medialis
**mAMEP**	m. adductor mandibulae externus profundus
**mAMES**	m. adductor mandibulae externus superficialis
**mAMP**	m. adductor mandibulae posterior
**mIRA**	m. intramandibularis
**mPSTs**	m. pseudotemporalis superficialis
**mPTd**	m. pterygoideus dorsalis
**mPTv**	m. pterygoideus ventralis
**ptb**	pterygoid buttress
**ptf**	pterygoid flange
**te**	tendon
**tr**	trochlea

## Materials and Methods

Ten alligator heads were obtained from the Rockefeller Wildlife Refuge, Grand Chenier, Louisiana including those from a large individual (AL 22, skull length [SL] 300.31 cm), five juveniles (18 cm−12.8 cm SL), three yearlings (∼6 cm SL), and one late term embryo (3.22 cm SL; Ferguson Stage 28, [15]). Specimens were either fresh frozen or formalin-fixed prior to study. Specimen identification and use is summarized in Table 2.

**Table 2:** Alligator specimens and techniques used to investigate cartilago transiliens anatomy.

Specimen Number	Skull Length (mm)	Imaging	Histology
**AL 114**	32.2 (Embryo)	μCT	Parasagittal/Axial
**AL 30**	54.3		Parasagittal
**AL 31**	58.9	μCT	N/A
**AL 32**	56.6	μCT in I_2_KI	N/A
**AL 03**	127.8	CT	Parasagittal/Horizontal
**AL 06**	155.4	CT	Parasagittal/Axial
**AL 34**	164.68	CT	Parasagittal/Horizontal
**AL 19**	178.2	N/A	Parasagittal
**AL 04**	189.5	CT	Parasagittal/Horizontal
**AL 22**	300.31 (Adult)	CT/MRI	N/A

### Macroscopic assessment

**Figure 1:**
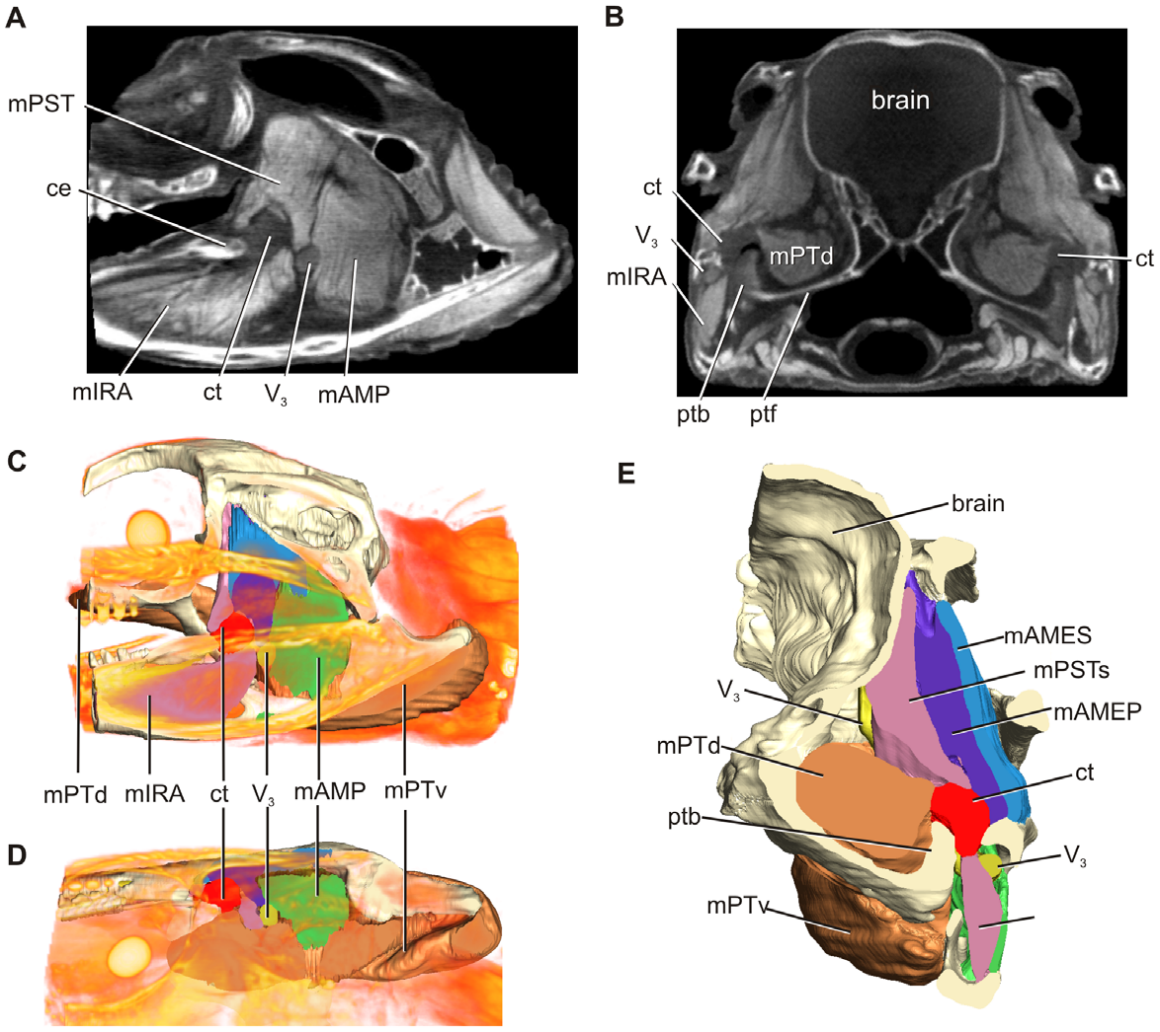
Lugol's iodine-stained μCT-scanned yearling alligator. **A**, Parasagittal section. **B**, Axial section. The μCT data were used to reconstruct 3D surface models using Amira 5.2 to illustrate the cartilago transiliens, muscles, and nerve. **C**, Left, lateral view. **D**, Mirrored, dorsal view. **E**, Rostral view. Muscles are color-coded to reflect hypotheses of jaw muscle homology described in Holliday and Witmer [13] and Holliday [27] and consistently used throughout the remainder of figures. The 3D model of alligator jaw muscles is available on the Holliday lab website.

One adult and three juvenile specimens were scanned on a medical GE LightSpeed VCT computed tomography scanner (0. 625 cm slice thickness), enabling visualization of skeletal elements, the cartilago transiliens, and some of the latter's surrounding tissues. The largest specimen was also scanned using a 3-Tesla Siemens Trio Magnetic Resonance Scanner at the University of Missouri Brain Imaging Center, which enables good visualization of soft tissue structure and some muscle fiber orientation. Finally, one yearling alligator head was fixed and saturated with Lugol's Iodine (I_2_KI) using techniques modified from that described by Metscher [16] and Jeffrey et al., [17]. The specimen was then scanned on a Siemens Inveon MicroCT scanner at a slice thickness of 83 microns. This process allowed excellent visualization of soft tissue. All scans were imported as DICOM files into Amira v5.2 (Visage Imaging) for segmentation and analysis. In addition to skull elements, soft tissue structures, specifically the temporal muscles m. pseudotemporalis superficialis (mPSTs), m. adductor mandibulae posterior (mAMP), m. intramandibularis (mIRA), m. adductor mandibularis externus profundus (mAMEP), m. adductor mandibularis externus superficialis (mAMES), m. pterygoideus dorsalis (mPTD), m. pterygoideus ventralis (mPTv), the mandibular nerve (V_3_), as well as the cartilago transiliens, were segmented for qualitative analysis of cartilago transiliens shape and topology with respect to neighboring structures (Fig. 1). The 3D model of Alligator jaw muscles used in this paper will be made available on the Holliday lab website.

### Dissection and Histology

Relevant regions of the embryo, a yearling, and two medium sized juveniles were dissected by hand and photographed for visualization of muscle attachments, tendinous insertions, and macroscopic fiber orientation using either a Nikon D90 DSLR camera or a Nikon SMZ-1000 Dissection microscope fitted with a DS-Fi1 digital camera. The right and the left cartilago transiliens' were dissected from all ontogenetic stages assessed except the large adult. Orientation of the cartilage was noted and marked via tying sutures to the dorsal and anterior sides of the cartilage prior to embedding. Once excised, the cartilago transiliens' were fixed in 10% neutral buffered formalin and then decalicified. Cartilages were variably sectioned prior to embedding in order to access the deeper portions the tissues. Typically, each left cartilage was sectioned dorsoventrally (parasagittally) and the right cartilage sectioned rostrocaudally (axially). Each half-unit was embedded in paraffin, serially-sectioned on a rotary microtome, and mounted on glass slides. Slides were alternately stained using Hematoxylin & Eosin, Masson's Trichrome, or Picrosirius Red (PSR) in order to study the cartilage, muscle, collagen fibers and other tissues of the cartilago transiliens in the cross-sectional sample of alligators. Slides were viewed and photographed using either an Aperio Scanscope CS scanner with ImageScope software, or an Olympus BX41TF microscope with an Olympus DP71 camera. Unstained and PSR-stained slides were also studied using an Olympus CX3/PF Polarizing microscope with Pixelink camera.

## Results

### Sesamoid anatomy

**Figure 2:**
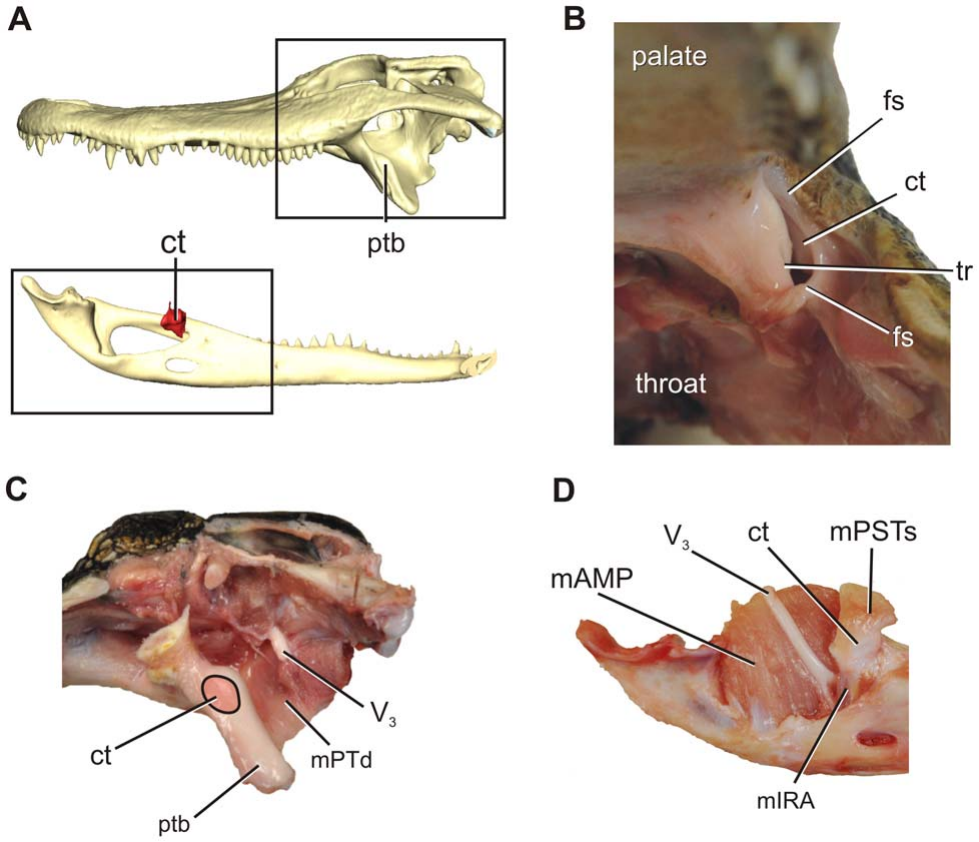
Gross anatomy of the pterygoid buttress and cartilago transiliens. **A**, 3D reconstruction of skull in lateral view and mandible in medial view, showing position of the pterygoid buttress and cartilago transiliens respectively. **B**, Cranial view of the rictal region. The mandible is slightly everted laterally. The cartilago transiliens is bounded laterally by skin and muscles, dorsally and ventrally by fibrous sheaths, and medially it articulates with the pterygoid buttress. **C**, Grossly dissected adductor region (box in A). The green oval shape denotes the resting position of cartilago transiliens on the rostrodorsal-most part of the pterygoid buttress trochlea. **D**, Dissected mandibular fossa with cartilago transiliens in situ.

In young alligators, the cartilago transiliens is situated at the caudolateral edge of the pterygoid buttress (Fig. 2). The cartilago transiliens is bounded laterally by skin and the rostralmost fibers of mAMES, and both dorsally and ventrally by fibrous sheathes such that when the mandible is everted laterally from the median plane, an oval-shaped hiatus is visible between the dorsal, lateral, and ventral boundaries of cartilago transiliens (Fig. 2B). The rostromedial surface of the fibrous capsule is continuous with the fibrocartilaginous core, which exhibits a shallow, saddle-shaped facet that articulates with the trochlear surface of the pterygoid buttress (Fig. 2D). The fibrous capsule attaches laterally onto the medial surface of the fibrocartilage-covered coronoid eminence of the mandible via the coronoid aponeurosis. Manipulation of the mandible in thawed specimens shows that the cartilago transiliens capsule slides along the pterygoid trochlea rostrodorsally-caudoventrally, but with limited movements in respect to the mandible. This suggests the coronoid aponeurosis constrains excursions of the cartilago transiliens against the coronoid during jaw movement. On macerated specimens, the coronoid eminence exhibits a distinct, rugose surface, indicative of its fibrocartilage covering during life.

### Neuromuscular anatomy

**Figure 3:**
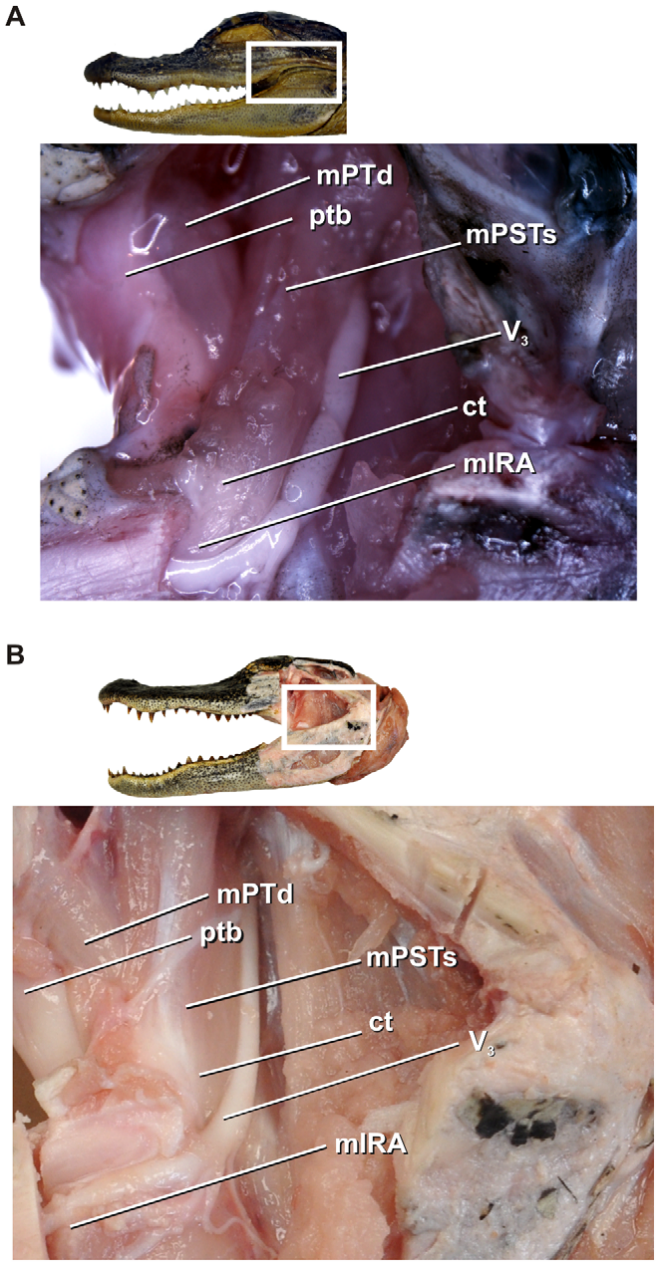
Cartilago transiliens and adductor chamber soft tissues maintain topological similarity through ontogeny, left lateral view. **A**, Yearling; **B**, Juvenile alligator.

The fibrous capsule of the cartilago transiliens attaches to several neighboring soft and bony structures. The dorsolateral surface of fibrous capsule attaches to M. adductor mandibulae externus profundus via an aponeurosis. M. adductor mandibulae externus profundus originates on the lateral surface of the parietal and rostral surface of the squamosal (which forms the rostrolateral border of the dorsotemporal fenestra), while inserting onto the rostralmost portion of the coronoid eminence on the lower jaw [13]. The dorsal portion of the fibrous capsule receives the ventral tendinous insertion of mPSTs, a muscle that originates on the lateral surface of the laterosphenoid, ventral to the dorsotemporal fenestra. Ventrally, the fibrous capsule of the cartilago transiliens serves as the origin of mIRA, which then inserts deep into the Meckel's fossa of the mandible. Medially, the capsule attaches to mPTd via an aponeurosis which extends onto the muscle surface. Caudally and ventrolaterally, the capsule is bounded by the mandibular nerve (CN V_3_), which passes lateral to mPSTs and mIRA as it enters the mandible. As suggested by [13], the relative topological position of the cartilago transiliens and its surrounding neuromusculature are consistent across ontogenetic stages (Fig. 3). Both mPSTs and mIRA receive motor innervation from branches of the mandibular nerve; however these branches are separated significantly from one another as those innervating mPSTs are found far more proximally on the mandibular nerve. Although topological positions of muscles and nerves have proven to be strong criteria for homology, their motor innervation has not [13].

### Microanatomy

**Figure 4:**
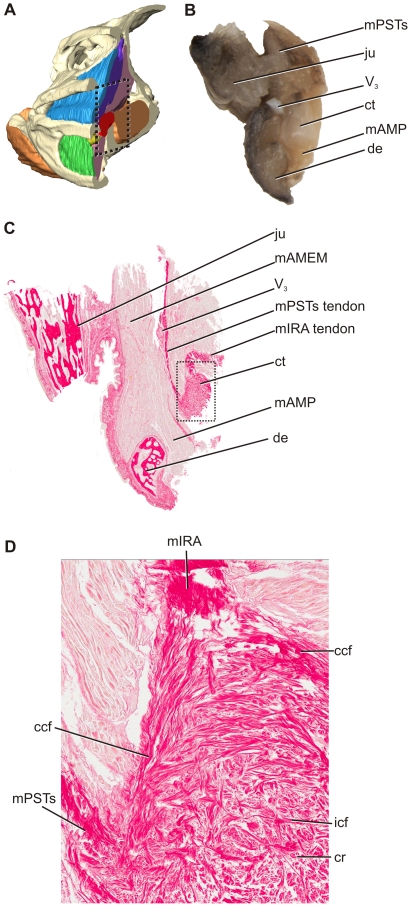
Anatomy of the alligator rictal region, showing the relative position of adductor musculature, cartilago transiliens, bone, nerve. **A**, Reconstructed model of Lugol-stained yearling μCT, coronally sliced. **B**, Dissected rictal region of embryonic alligator in right, cranial view. **C**, coronal histological section of the same region, in cranial view with inset for D. **D**, The fibrocartilaginous nodule is composed mostly of unorganized internal collagen fiber woven within the cartilage matrix. Continuous collagen fibers pass laterally to the fibrocartilaginous nodule, bridging m. pseudotemporalis superficialis tendon to m. intramandibularis tendon. Note: the cartilago transiliens in the specimen became rotated counter- clockwise during preparation leading to the change in orientation in mPSTs and mIRA.

**Figure 5:**
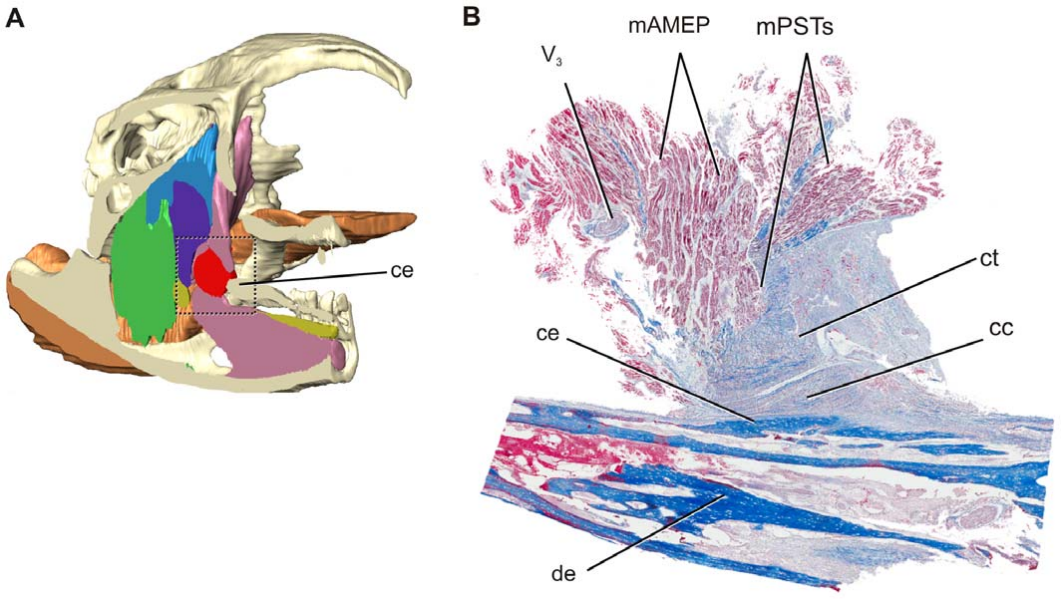
Right, parasagittal histological section of cartilago transiliens tendon organ from a late term embryo in lateral view. Section was taken on the lateral edge of the cartilaginous transiliens to demonstrate superficial muscle and tendinous attachments. The m. intramandibularis tendon is obscured from view by the dentary.

Histology of the cartilago transiliens in the late-stage embryo shows that the structure arises as a fibrous swelling within the medial part of the m. pseudotemporalis superficialis tendon. At this stage, coronal section of the embryonic cartilago transiliens shows clear continuity of collagen fibers of mPSTs and mIRA surrounding a bulging, central nodule of fibrocartilage (Fig. 4C). In the embryonic cartilago transiliens, the fibrocartilaginous core is composed of disorganized collagen fibers interwoven within the cartilage matrix (Fig. 4D). Parasagittal section of the embryonic cartilago transiliens shows the presence of strong, rostrally-directed collagen fibers, similar in position with those of the coronoid aponeurosis observed by gross dissection of older individuals. Ventrolaterally, the cartilago transiliens borders a distinct, separate pad of fibrocartilage on the coronoid eminence of the mandible. Rostrally, the embryonic cartilago transiliens is composed primarily of dorsoventrally oriented, investing collagen fibers from mPSTs (Fig. 5). These fibers showed parallel arrangement and are more tightly packed than those found at the core. In addition, the embryonic cartilago transiliens has caudodorsolateral attachment of mAMEP. However, this attachment appears only superficial, with no tendon or collagen fibers continuing into the fibrocartilaginous matrix of the cartilago transiliens.

**Figure 6:**
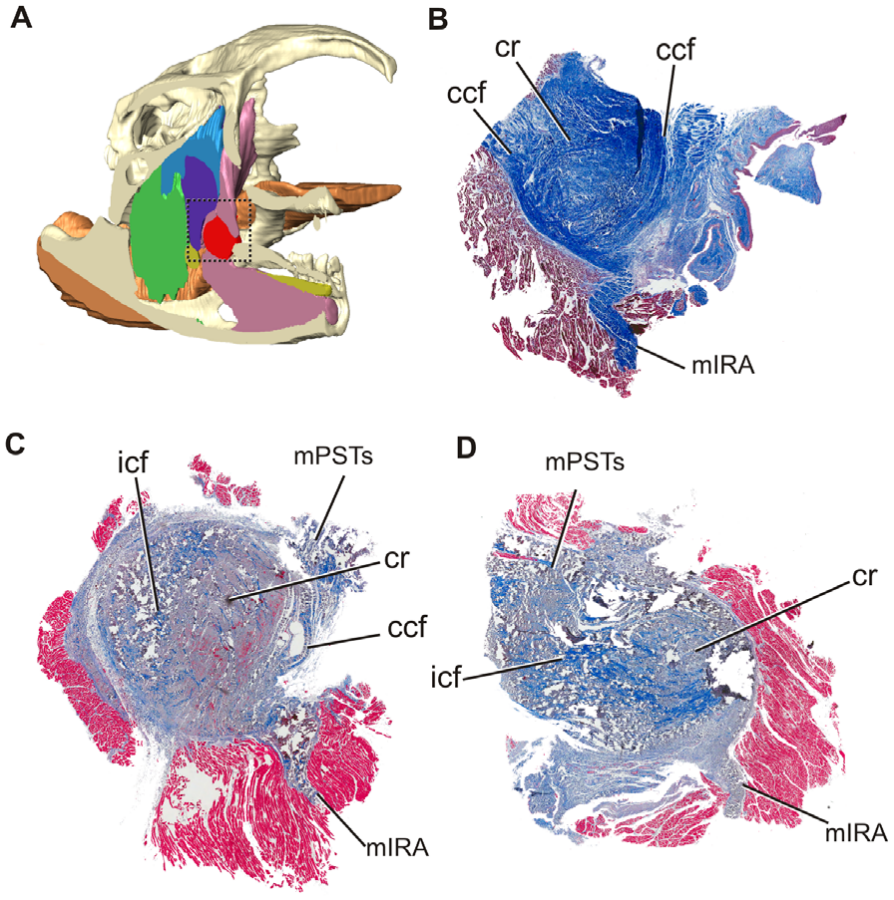
Right, parasagittal sections of the cartilago transiliens. All sections are arranged dorsal to the top and cranial to the right. **A**, Reconstructed model of Lugol-stained yearling μCT, parasagittally sliced to show cartilago transiliens and adnexa. **B**, Lateral section from yearling. **C**, Medial section of cartilago transiliens from small juvenile. **D**, Lateral section of cartilago transiliens from a subadult.

**Figure 7:**
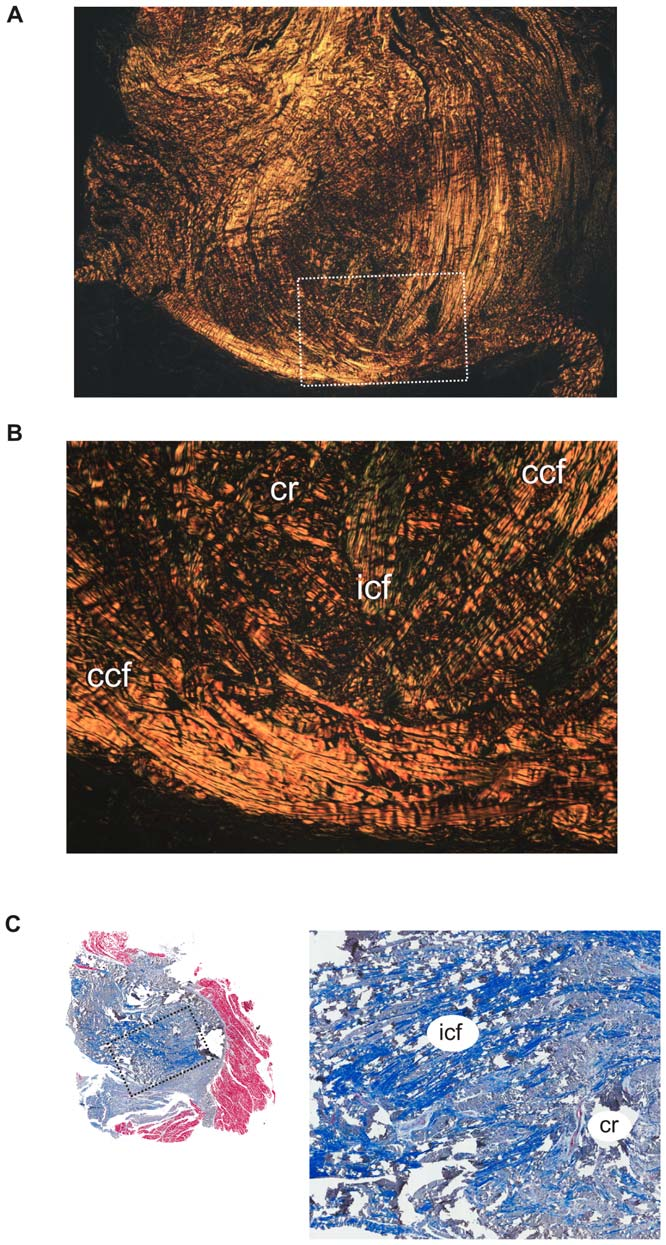
Ontogenetic differences between parasagittal histological sections of cartilago transiliens of a yearling (PSR, polarized light) and a subadult (Masson's trichrome). The boxes correspond to higher magnification views. **A**, In the yearling, collagen fibers from mIRA tendon are continuous with mPSTs, encircling the fibrocartilaginous nodule both rostrally and caudally. **B**, Higher magnification of inset in A. **C**, In a juvenile, collagen and muscle fiber from mPSTs still inserts onto the fibrocartilaginous nodule. However, in the core regions of the cartilago transiliens (enlarged picture to right), collagen fibers show no continuity between mPSTs and mIRA tendons.

Parasagittal sections of the yearling's cartilago transiliens (Fig. 6) show that it is also composed mostly of vertically oriented, continuous collagen fibers from mPSTs and mIRA. Continuous collagen fibers surround the nodule on both rostral and caudal sides, whereas internal collagen fibers become more organized, parallel to the direction of the continuous collagen fibers. Visualization under polarized light (Fig. 7A) also shows that the continuous collagen fibers separate the cartilaginous nodule from the surrounding muscles. Horizontal sections of the yearling specimen also show incipient organization of collagen fibers within the cartilago transiliens, with the medial portion composed mostly of cartilage, and the lateral portion mostly of rostrocaudally or dorsoventrally oriented continuous collagen fibers.

**Figure 8:**
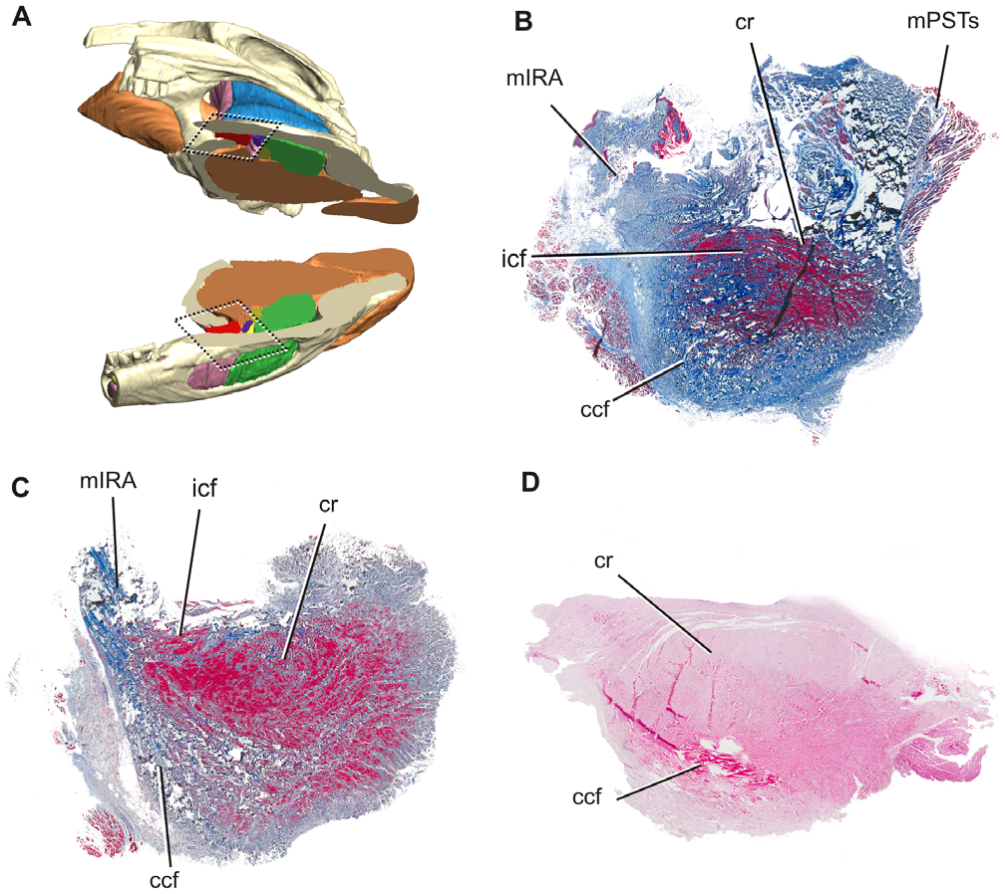
Ontogenetic differences between horizontal sections of cartilago transiliens. All sections are arranged medial to the top and rostral to the right. **A**, Reconstructed model based on Lugol-stained yearling µCT, horizontally sliced. **B**, Dorsal section of ct from small juvenile; **C**, Ventral sections of ct from a medium-sized juvenile. **D**, Dorsal section of a medium-sized juvenile.

**Figure 9:**
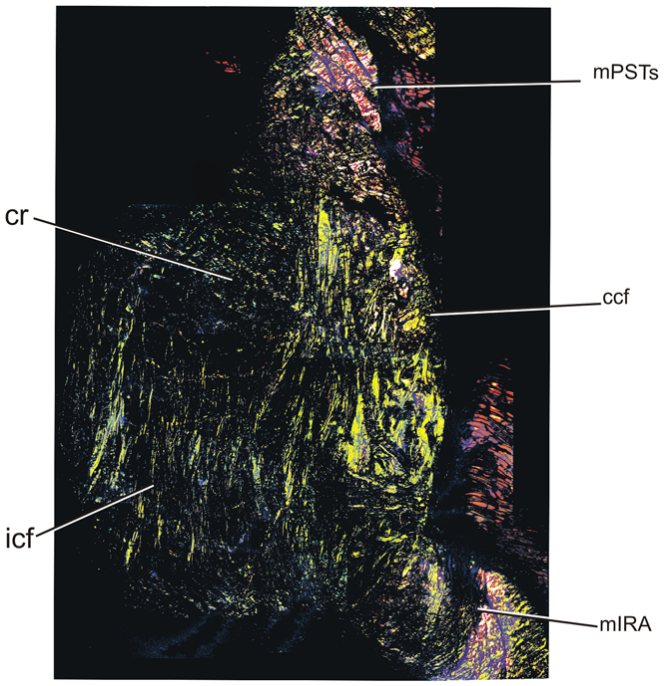
Collagen fiber orientation in a left parasagittal section of the cartilago transiliens of a juvenile under polarized light illustrating the continuity of fibers between mIRA and mPSTs caudal to the nodular, cartilaginous region.

In a small juvenile, the cartilago transiliens showed further specialization as a sesamoid. Horizontal sections showed that in addition to the further increase in relative proportion of the cartilaginous matrix in the medial part of the cartilago transiliens, collagen fibers from mPSTs and mIRA now remain continuous only at the most lateral portion of the fibrocartilaginous nodule (Fig. 8). The caudomedial periphery of the cartilago transiliens retains some remnant of the once parallel, organized collagen fibers, but the fibers are now thinner and appeared to be interwoven in a large portion of the cartilaginous matrix (Fig. 9). Chondrification of the rostromedial periphery appeared most extensive. In parasagittal view, outermost collagen fibers within the fibrocartilage shows continuity with vertically oriented collagen fibers from the mPSTs and mIRA on both rostral and caudal aspects of the nodule. However, the rostral bundle appears relatively less extensive than the caudal ones (Fig. 6).

**Figure 10:**
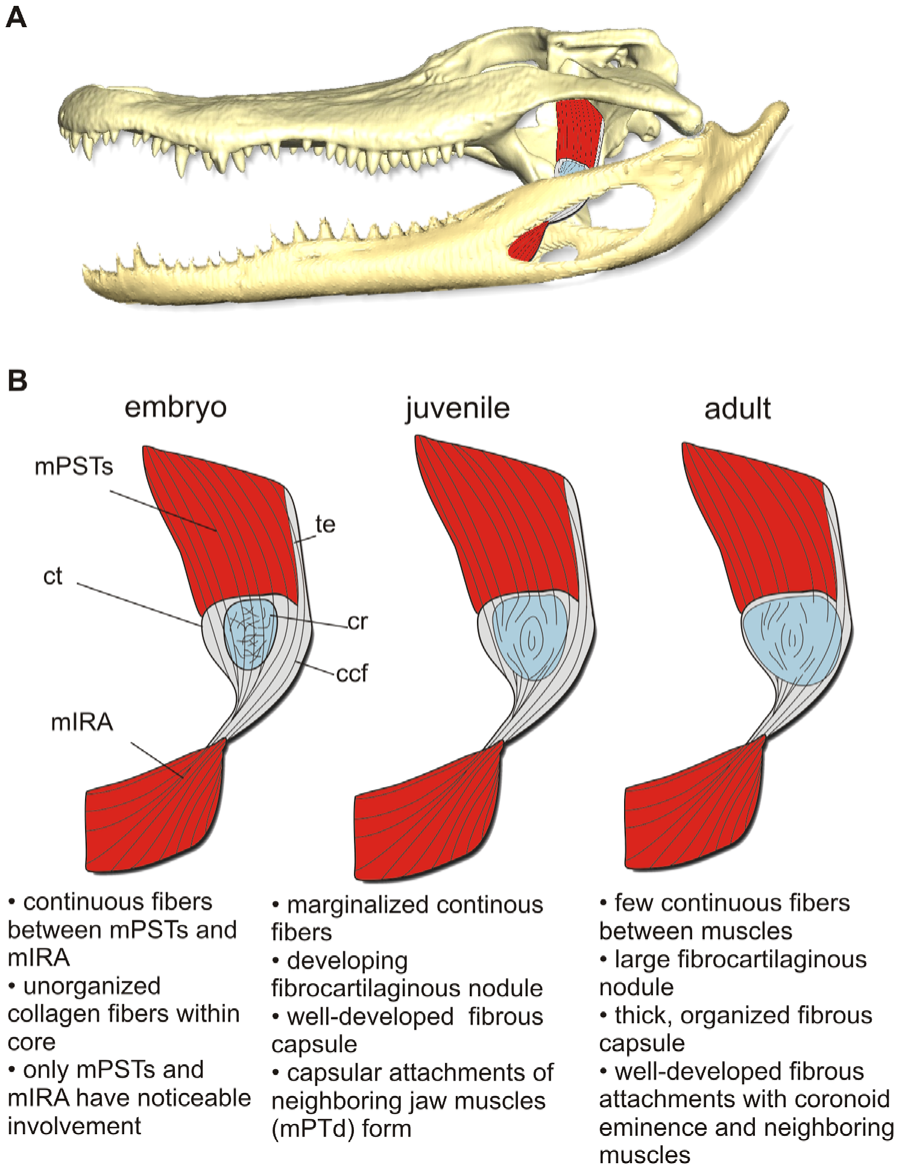
The key ontogenetic changes of the cartilago transiliens tendon organ in *Alligator mississippiensis*. **A**, Topological position of the cartilago transiliens in lateral view. **B**, Illustrations depicting morphological changes and characteristic differences among differently-aged individuals.

In the juvenile individuals, the cartilaginous core composed a much greater portion of the cartilago transiliens than in younger ontogenetic stages (Figs. 6, 8). Parasagittal sections showed that collagen fibers inside the nodule are more organized, circumferentially traversing within the nodule's matrix (Fig. 7). Collagen fibers from mPSTs still exist on the dorsal periphery of the cartilaginous nodule, but continuity of these fibers with mIRA could no longer be observed. Horizontal sections of juveniles show that organized, parallel collagen fibers exist mostly in the lateral side of the cartilago transiliens complex, whereas the medial side composed mostly of cartilaginous matrix interwoven with unorganized collagen fibers (Fig. 9). Overall, as alligators age, the rostral collagen fiber bundle decreases in continuity between the mPSTs and mIRA (Fig. 10B).

## Discussion

As defined by Vickaryous and Olson [2], a sesamoid is a small concentration of skeletal tissue shrouded within regular dense connective tissue, located proximate to a bony prominence over which the connective tissue wraps. Gross anatomical and histological analysis show that the crocodylian cartilago transiliens fits this definition and is not a novel, solitary soft-tissue structure as typically described. The cartilago transiliens complex is present in all individuals evaluated, with no noticeable topological changes of the cartilage and its neighbors through ontogeny. The cartilago transiliens' gross anatomy in crocodylians shows similarities with the analogous “os”, or “cartilago transiliens” intertendon in turtles [18], [12], in which the fibrocartilage tendon organ occurs within the mAME complex passes over hyaline-cartilage covered trochlea on the quadrate or prootic. These insights into the crocodylian cartilago transiliens further suggest that the fibrocartilaginous intertendon between mPSTs and mIRA of birds and turtles noted by Holliday and Witmer [13] may also be a similar, albeit, less nodular structure, thus suggesting these muscles may also be evolutionarily similar and possibly homologous although additional developmental details are needed to test this hypothesis.

The microstructure of the cartilago transiliens resembles that of the fibrocartilaginous pad located inside the adductor mandibularis medialis muscle of the cownose ray [19]. In both structures, the cartilaginous region of the fibrocartilage is medially differentiated from fibrous, muscular regions. In these fibrous regions, collagen fibers occur in organized bundles and run continuously with the wrap-around tendon; whereas the cartilaginous regions exhibit a predominance of cartilaginous matrix interlaced with non-linearly oriented collagen fibers, at articulation sites with the hard-tissue trochlea, where compressive force is expected to be the greatest. The cartilago transiliens shows microstructural similarities to the sesamoid fibrocartilage associated with human retrocalcaneal bursa described by Shaw et al [3] in which the incompressible fibrocartilaginous nodule forms during embryological development well before the wrap-around tendon experienced rigorous compression from ex-utero joint movement. Despite their utility in understanding biomechanical and evolutionary patterns in muscles, such as the use of cranial sesamoids as characters in understanding catfish evolution [22], the term “sesamoid” is often used as a wastebasket term for accessory skeletal elements [2]. But, here we see that among the jaw muscles of vertebrates, these intertendons, fibrocartilaginous pads, and sesamoids appear to only differ in the extent of their nodular morphology while maintaining very similar gross and histological structure. This suggests not only that these structures are all simply part of the same spectrum of intramuscular connection, but also that the differently-named muscle bellies connected by intertendons, pads, nodules, or sesamoids (again, here argued as different names for largely the same structures) may actually be the same muscle. Although these hypotheses of homology require additional testing at numerous levels of similarity (e.g., phylogenetic, developmental, topological), the evidence provided here indicates that at least in crocodylians, m. intramandibularis, the cartilago transiliens enthesis organ, and m. pseudotemporalis superficialis are likely all one muscular unit.

The continuity of collagen fibers from mPSTs and mIRA decreased as the fibrocartilaginous, nodular core of the cartilago transiliens enlarged during ontogeny. During early ontogeny, the cartilaginous matrix began forming at the central core of the cartilago transiliens, with continuous fibers on both rostral and caudal boundaries of the cartilaginous nodule. Yet, the rostromedial edge of the fibrocartilage showed fewer parallel, continuous collagen fibers from mPSTs and mIRA, instead exhibiting more cartilage matrix interwoven with only a few internal fibers among older individuals. Macroscopic observation showed that the articulation facet of the cartilago transiliens is situated on its rostromedial surface (Fig. 2). The presumably highest level of mediolateral compression upon mPSTs against the pterygoid trochlea at this site would be consistent with its elevated rate of chondrification similar to Summers' [19] description of the hyaline-like cartilage in fibrocartilage ossicles within sesamoids of wrap-around cranial joints in elasmobranchs. The differential microstructure at various sites is reflective of different mechanical demands placed upon the structure, with continuous, vertical collagen fibers on the lateral aspects best suited for axial tension and the cartilaginous regions more resilient to mediolateral compressive loads [4]. Hence, our histological data suggest that the cartilago transiliens is a sesamoid.

All extant crocodylians possess cartilage-covered trochlear surfaces on the lateral surface of the pterygoid buttress, as well as rugosities on the coronoid eminence [20]. As noted by Benjamin and Ralphs [5], such surfaces are closely associated with fibrocartilaginous sesamoids. Large, descending pterygoid buttresses with a trochlear surface are ubiquitous among extinct crocodyliforms [21], [23, pers. obs.] indicating that the cartilago transiliens was likely a ubiquitous feature among crocodyliforms. Schumacher [12], Iordansky [10], and Van Drongelen and Dullemeijer [14] all described how, during jaw opening, mPSTs and the cartilago transiliens are stretched over the pterygoid buttress trochlea as the direction of the muscle changes from being obliquely horizontal to near vertical where mIRA attaches within the mandibular fossa. Therefore, the cartilago transiliens must experience significant compression against the buttress during jaw opening and feeding behaviors. Finite element modeling of the alligator mandible indicates that the pterygoid buttress indeed stabilizes the mandible against mediolateral bending during jaw closing [24], therefore creating a highly compressive environment for any soft tissues lying between the two bony elements. Thus, the biomechanical environment suits the formation of a sesamoid, and the cartilago transiliens develops exactly at the site of compression between mandible and the pterygoid buttress. Therefore, in biomechanical sense, the cartilago transiliens is likely a sesamoid.

Extant crocodylians are characterized by dorsoventrally flattened skulls. Consequently, the insertion site of the mPSTs in the mandibular fossa is shifted laterally to its origin on the laterosphenoid, thus increasing the medial component of the contractile force generated by mPSTs. It is likely that in extant crocodylians, the cartilago transiliens-pterygoid buttress tendon organ is used as a pulley system that redirects muscular forces of mPSTs more vertically as it inserts into the lower jaw [10]. In addition, Dullemeijer and Van Drongelen [14] argued that the cartilago transiliens functions in extant crocodylians as a jaw-locking mechanism. Their electromyographical studies confirmed that adductor muscle contractions occur well before jaw movement, and attributed this observation to the cartilago transiliens' resistance of muscular force. Certainly, such resistance would allow modern crocodylians to exert less muscular force for holding their jaws open while basking, or closed while holding onto and submerging struggling prey. However, we think it is unlikely that the cartilago transiliens-pterygoid buttress tendon organ evolved specifically as a structure for the specific functional demands of jaw locking, as the pterygoid buttress maintains several different shapes, sizes, and relative positions across a wide range of extinct crocodyliforms with a variety of skull shapes and ecological roles, such as terrestrial predators [23], terrestrial herbivores [21] and fully marine taxa [25]. Although a more in-depth understanding of the evolution of the pterygoid flange-mandible system of sauropsids is required, these biomechanical and evolutionary patterns suggest the buttress primarily evolved to brace the mandible [24] and then later facilitated a jaw locking behavior. Using modeling techniques, Goessling et al., [26] hypothesized the cartilago transiliens acts as a means to achieve equilibrium of jaw muscle forces and moments, a function that would likely supersede jaw locking behavior. Overall, this research on the ontogeny of the cartilago transiliens of crocodylians suggests that the jaw adductor muscles mPSTs and mIRA are the same muscle, and that the cartilago transiliens evolved as a sesamoid to resist mediolateral compressive forces imposed on the mPSTS tendon as the latter wraps around the pterygoid buttress.
